# T Cell Epitope Immunotherapy Induces a CD4^+^ T Cell Population with Regulatory Activity

**DOI:** 10.1371/journal.pmed.0020078

**Published:** 2005-03-29

**Authors:** Adrienne Verhoef, Clare Alexander, A. Barry Kay, Mark Larché

**Affiliations:** Department of Allergy and Clinical Immunology, Imperial College LondonNational Heart and Lung Institute, LondonUnited Kingdom; University of VirginiaUnited States of America

## Abstract

**Background:**

Synthetic peptides, representing CD4^+^ T cell epitopes, derived from the primary sequence of allergen molecules have been used to down-regulate allergic inflammation in sensitised individuals. Treatment of allergic diseases with peptides may offer substantial advantages over treatment with native allergen molecules because of the reduced potential for cross-linking IgE bound to the surface of mast cells and basophils.

**Methods and Findings:**

In this study we address the mechanism of action of peptide immunotherapy (PIT) in cat-allergic, asthmatic patients. Cell-division-tracking dyes, cell-mixing experiments, surface phenotyping, and cytokine measurements were used to investigate immunomodulation in peripheral blood mononuclear cells (PBMCs) after therapy. Proliferative responses of PBMCs to allergen extract were significantly reduced after PIT. This was associated with modified cytokine profiles generally characterised by an increase in interleukin-10 and a decrease in interleukin-5 production. CD4^+^ cells isolated after PIT were able to actively suppress allergen-specific proliferative responses of pretreatment CD4^neg^ PBMCs in co-culture experiments. PIT was associated with a significant increase in surface expression of CD5 on both CD4^+^ and CD8^+^ PBMCs.

**Conclusion:**

This study provides evidence for the induction of a population of CD4^+^ T cells with suppressor/regulatory activity following PIT. Furthermore, up-regulation of cell surface levels of CD5 may contribute to reduced reactivity to allergen.

## Abstract 2

Immunotherapy is one approach to treating cat allergy and asthma. One mechanism of action might be that it induces a population of CD4 positive T cells with suppressor activity

## Introduction

The central role of T cells in the pathogenesis of allergic disease is well established [1]. Through production of interleukin (IL)-4, IL-5, and IL-13, allergen-specific T helper (Th) 2 cells direct IgE synthesis, eosinophil growth/differentiation, and induction of airway hyperreactivity [2,3].Until recently, it was assumed that the basis for allergic disease was an imbalanced Th cell response to certain allergens, manifest as a predominance of Th2 cytokines over Th1 cytokines. However, immune suppression may also be a normal consequence of a protective immune response, serving to limit excessive responses that lead to immunopathology [4]. The role of regulatory T cell (T_reg_) populations in maintaining homeostasis is increasingly well understood. The term T_reg_ is used to describe a variety of T cell functional phenotypes that display common features. Several studies have described the dependence of T_reg_ function on cell–cell contact. In certain cases regulation was demonstrated to be dependent on IL-10 and/or transforming growth factor β secretion [5,6,7,8,9].

Regulation of immune responses may be attributable to both naturally occurring (thymus-derived, or “natural”) regulatory cells and also naïve or effector T cells that have acquired suppressive activity (adaptive regulatory cells) [10,11]. Therapeutic administration of short, soluble peptide sequences, in the absence of inflammatory signals, may result in presentation by immature or quiescent antigen-presenting cells (APCs). Immature allogeneic human dendritic cells (DCs) induced non-proliferating, IL-10-producing CD4^+^ T cells with regulatory properties [12], while peptide-specific human T_reg_ were induced following administration of antigen-pulsed immature DCs in vivo [13,14]. DCs producing IL-10 were able to suppress airway inflammation in a murine model of asthma [15]. Thus, partially immature or “steady state” DCs, circulating in the lymphatics, may interact with T cells in a tolerogenic milieu, in the absence of concomitant pro-inflammatory stimuli such as pattern recognition receptor triggering [16].

An additional mechanism for limiting immune responses may be reducing sensitivity to cognate signals. Up-regulation of CD5, a suppressor of T cell signalling [17], has been associated with regulatory cells arising as a consequence of competition for space and resources [18]. Under such conditions, suppression was shown to lack antigen specificity and to be mediated by cells that did not exhibit any of the hallmarks of “professional” T_reg_. Recently, Hawiger and colleagues delivered antigen to steady-state DCs via the DEC-205 molecule. Following cognate interaction with these cells, antigen-specific T cells were unresponsive and expressed enhanced levels of CD5 [19]. Chronic low-level antigen exposure in the periphery has also been shown to result in anergy in CD8^+^ cells that was associated with increased expression of CD5, further illustrating a role for CD5 in regulation of T cell function [20].

In animal models, the administration of low-dose peptide is a well-established mechanism for the induction of T_reg_ that may arise as a result of presentation by steady-state DCs and “non-professional” APCs [21,22,23,24]. Similarly, administration of soluble peptides to allergic asthmatic individuals has been shown to result in markedly reduced cutaneous reactions to allergen injection [25,26,27], reduced airway hyperreactivity [27], and improvements in symptom scores after nasal allergen challenge [28]. Changes in clinical reactivity were associated with decreased Th1 and Th2 cytokines and increased IL-10 production [25,26]. In the current study, we address the hypothesis that low-dose peptide therapy in allergic individuals results in antigen-specific hyporesponsiveness associated with the induction of a suppressive population of CD4^+^ T cells, together with up-regulation of surface CD5 levels on antigen-specific T cells.

## Methods

### Patients and Study Design

Individuals who were cat-allergic and asthmatic were recruited, diagnosed, and assessed as described in detail elsewhere [29]. The study received prior approval from the Ethics Committee of the Royal Brompton and Harefield Hospitals National Health Service Trust (London, United Kingdom). Written, witnessed informed consent was obtained from all patients. Peripheral blood mononuclear cells (PBMCs) were obtained from patients enrolled in two consecutive studies (open study design) of immunotherapy employing short synthetic peptides derived from the sequence of the major cat allergen Felis domesticus allergen 1 (Fel d 1). The studies employed different dosing regimes in order to evaluate dose effects on clinical and mechanistic outcomes. The first study included eight patients (referred to hereafter as Group 1) who received incremental doses of Fel d 1 peptides (0.1, 1, 1, 5, 10, and 25 μg) totalling 42.1 μg of each peptide, while the second study comprised 12 patients (referred to hereafter as Group 2) who received a total of 291 μg (1, 5, 10, 25, 50, 100, and 100 μg) of each peptide. Peptides were synthesised by Fmoc chemistry, purified by HPLC, and presented as lyophilised solids (Advanced Biotechnology Centre, Imperial College London, United Kingdom). Peptides were reconstituted with sterile physiological saline and dispensed into sterile vials for single patient use (Nova Laboratories, Leicestershire, United Kingdom). Peptide sequences were as follows: EICPAVKRDVDLFLTGT, LFLTGTPDEYVEQVAQY, EQVAQYKALPVVLENA, KALPVVLENARILKNCV, RILKNCVDAKMTEEDKE, KMTEEDKENALSLLDK, KENALSLLDKIYTSPL, LTKVNATEPERTAMKK, TAMKKIQDCYVENGLI, SRVLDGLVMTTISSSK, ISSSKDCMGEAVQNTV, and AVQNTVEDLKLNTLGR.

Clinical parameters and outcome measures associated with peptide intervention in donors from whom PBMC samples were obtained are described in detail elsewhere [27,28]. Briefly, in the first study peptide immunotherapy (PIT) resulted in improved non-specific bronchial hyperreactivity, since a significantly (*p =* 0.02) greater concentration of histamine was required to induce a 20% reduction in forced expiratory volume measured in 1 s. Additionally, a significant reduction (*p =* 0.03) in the magnitude (area in square millimeters) of the late-phase skin reaction was observed post-treatment. In the second study, treatment was associated with a reduction in the magnitude of the late asthmatic reaction induced by inhaled allergen challenge, together with a significant decrease in nasal outcome measurements (number of sneezes, nasal blockage, and weight of nasal secretion; all measurements at 15 min post-challenge, *p =* 0.02).

### PBMC Cultures

PBMCs were isolated from venous blood by density gradient centrifugation (Histopaque-1077; Sigma Chemicals, Poole, United Kingdom) and cryopreserved. All experiments were performed on pre- and post-PIT PBMCs of the same patient in single experiments, to reduce inter-experiment variation within single patients. Prior to in vitro culture, PBMCs were thawed, washed, and labelled with carboxyfluorescein diacetate succinimidyl ester (CFSE) (Molecular Probes, Eugene, Oregon, United States), as follows: 2.5 × 10^6^ each of pre-PIT and post-PIT PBMCs were resuspended in 0.5 ml of RPMI-1640 (Invitrogen, Paisley, United Kingdom), and 0.5 ml of 1 μM CFSE added under constant, gentle agitation, to achieve a final CFSE concentration of 0.5 μM. After 10 min, 1 ml of human AB serum (Sigma, Poole, United Kingdom) was added to terminate labelling, and cells were washed twice. Cells were resuspended at 2.5 × 10^6^ cells/ml of complete medium (RPMI-1640 supplemented with L-glutamine and 5% human AB serum) and plated at 5 × 10^5^ cells/well in 96-well flat-bottom culture plates (Nunc, Merck Eurolab, Lutterworth, United Kingdom) in 200 μl of final volume, under the following culture conditions: unstimulated, stimulated with 30 μg/ml whole cat allergen (generous gift of Leti Laboratories, Madrid, Spain) and stimulated with plate-bound α-CD3/α-CD28 (10/1 μg/ml; BD Pharmingen, Cowley, United Kingdom).

For suppression experiments, PBMCs were separated into CD4^+^ and CD4^neg^ populations. Limited quantities of peripheral blood were available from study patients. Therefore, for reasons of economy, CD4-depleted PBMCs (CD4^neg^) remaining after selection of CD4^+^ cells were used as target cells in all suppression assays. For each patient, 20 × 10^6^ each of pre- and post-PIT PBMCs were labelled with αCD4 magnetic beads (MACS; Miltenyi, Bisley, United Kingdom) and positively sorted to a mean purity of 94%. CD4^neg^ pre- and post-PIT cells were labelled with CFSE as described above, while CD4^+^ pre- and post-PIT T cells were labelled with PKH-26 (Sigma) as follows: cells were resuspended in diluent C (Sigma) at no more than 10^7^ cells/ml, and an equal volume of a PKH-26 dilution (1 μM) was added to reach a final concentration of 0.5 μM. After 2 min, the reaction was stopped with the addition of 1 ml of human serum, and cells were washed twice. Cells were cultured in the following combinations: pre- and post-PIT CD4^neg^ cells alone, pre-PIT CD4^neg^ plus pre- or post-PIT CD4^+^, and post-PIT CD4^neg^ plus pre- or post-PIT CD4^+^ (CD4^neg^ cells at 0.5 × 10^6^ cells/well and CD4^+^ cells at 0.125 × 10^6^ cells/well in 96-well flat-bottom tissue culture plates, to achieve a ratio of 4:1). All were cultured in the absence or presence of cat allergen (30 μg/ml) for 1 wk in a humidified incubator at 37 °C gassed with 5% CO_2_ in air.

### Flow Cytometry

To determine changes in the proliferation of T cell subpopulations associated with PIT, cells were recovered after 1 wk of culture, washed twice, and stained for 30 min at 4 °C with a combination of αCD4-PE + αCD8-Cy, or αCD45-PE. Isotype controls used were mouse IgG_2a_-PE, mouse IgG_1_-PE, and mouse IgG_1_-Cy. Mean fluorescence intensity (MFI) was determined by FACS (FACScan, BD Pharmingen) of at least 2 × 10^4^ events within the lymphocyte gate. In suppression experiments, the extent of proliferation was measured as above on the CFSE-labelled read-out population without additional antibody staining. Percentages of CD4^+^CD25^+^ T cells, CD4^+^CD5^+^ cells, or CD8^+^CD5^+^ cells were measured for unstimulated cells by double staining with αCD4-Cy + αCD25-FITC, αCD4-Cy + αCD5-PE, or αCD8-Cy + αCD5-PE. Isotype controls used were mouse IgG_1_-Cy and mouse IgG_1_-FITC (all antibodies were from BD Pharmingen).

### Cytokine Measurements

Culture supernatants of 100 μl were collected from wells 48 h after the start of culture. Cytokines were measured by cytometric bead array Th1/Th2 kit (BD Pharmingen) according to the manufacturer's instructions. A total of six cytokines were measured simultaneously. Data for IL-5, IL-10, and interferon (IFN)-γ are shown. Cytokine concentrations were determined using cytometric bead array analysis software (BD Pharmingen). The sensitivity of the assays was 2.4 pg/ml for IL-5, 2.8 pg/ml for IL-10, and 7.1 pg/ml for IFN-γ.

### Data Analysis

FACS cell surface data and CFSE–PKH-26 mixing experiment proliferation data were acquired with Cellquest (BD Pharmingen), and events within the live lymphocyte gate were interpreted using Winmdi 2.8 software (Scripps Research Institute, http://facs.scripps.edu/software.html). CFSE proliferation data of T cell subsets were acquired with Cellquest, and events within the CD4^+^, CD8^+^, or CD45RO^+^ gate analysed with the Proliferation Wizard module in ModFit LT software (Verity Software House, Topsham, Massachusetts, United States). Percentage proliferation is defined as the fraction of the starting population that has proliferated during the course of the experiment.

### Statistical Analysis

For statistical analysis data were analysed for normality using the Shapiro-Wilks test. Normally distributed data were analysed using the paired t-test (parametric). Non-normal data were analysed using the Wilcoxon signed rank test (non-parametric). Analysis was performed by an independent statistician (Turnstat, Reading, United Kingdom).

## Results

### PIT Results in the Inhibition of Cat-Allergen-Induced Proliferation of CD45RO^+^, CD4^+^, and CD8^+^ T cell Subsets

**Figure 1:**
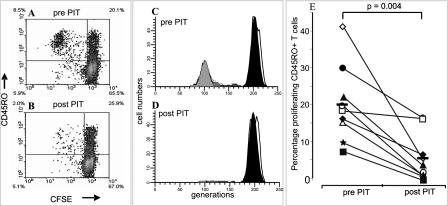
PIT Reduces Antigen-Specific Proliferation of Memory T Cells (A–D) PBMCs taken before and after PIT were labelled with CFSE to track cell division after antigen stimulaton. Proliferation of cat-allergen-specific CD45RO^+^ lymphocytes was reduced following PIT (A) and (B). (C) and (D) represent CD45RO^+^ T cells as shown in panels (A) and (B), respectively, analysed with ModFit software. The right-hand peaks represent the parental population, and generations of dividing cells are depicted leftwards along the x-axis. (E) Summary of the percentage of proliferating CD45RO^+^ T cells pre- and post-PIT (percent proliferating cells is defined as the fraction of the starting population that has proliferated during the course of the experiment, determined with Modfit) for all nine patients tested. Open symbols represent patients enrolled in treatment Group 1, while solid symbols depict patients from treatment Group 2. Horizontal solid bars show mean levels of proliferation. Background proliferation (in the absence of a stimulus) was less than 2% and was subtracted. The Wilcoxon signed rank test was used for statistical analysis.

The effect of PIT on cellular proliferation of T cell subsets was evaluated by combining CFSE labelling with cell surface staining. As shown in Figure 1A, the majority of cat-allergen-specific T cells resided within the CD45RO^+^ (memory) T cell population. The proliferation of this population was markedly inhibited following PIT (Figure 1A–1D). Limited allergen-specific proliferation was detected in the CD45RO^−^ (naïve) population, but this appeared less sensitive to the effects of PIT. Data from all nine individuals tested showed a similar reduction in the post-PIT proliferative response (Figure 1E; mean proliferation pre-PIT [20.3%] was decreased post-PIT [5.8%], *p =* 0.004; pre-PIT range 7.9%–41.8%; post-PIT range 0%–16.7%). Proliferation in the absence of a stimulus was less than 2% in all cases and was subtracted. Proliferation to plate-bound α-CD3/α-CD28 (10 μg/ml and 1 μg/ml, respectively, as a mixture) resulted in mean pre-PIT proliferation of CD45RO^+^ T cells of 64.2% and post-PIT proliferation of 60.1% (data not shown).

**Figure 2:**
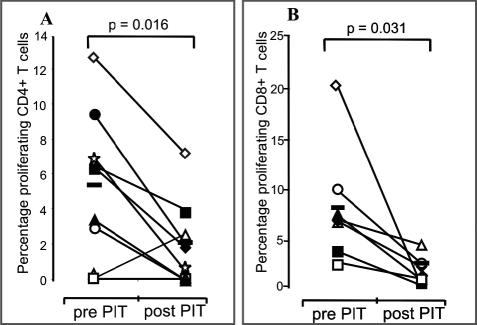
PIT Reduces Antigen-Specific Proliferation of CD4^+^ and CD8^+^ T Cells CD4^+^ and CD8^+^ proliferation data were obtained and interpreted as for Figure 1. (A) and (B) represent percentage proliferation of PBMCs to cat allergen for each patient as determined with ModFit. Open symbols represent patients from treatment Group 1, while solid symbols depict patients from treatment Group 2. Horizontal solid bars indicate means. Background proliferation has been subtracted. The Wilcoxon signed rank test was used for statistical analysis.

The effect of PIT on CD4^+^ and CD8^+^ populations was also addressed. Both CD4^+^ and CD8^+^ subsets proliferated to whole cat allergen. CD4^+^ post-PIT T cell proliferation was significantly reduced (*p =* 0.016; Figure 2A), despite a slight increase in proliferation for one patient (mean pre-PIT to post-PIT CD4 proliferation was reduced from 5.4% to 2.1% [pre-PIT range 0%–12.7%; post-PIT range 0%–7.3%]). A similar reduction was observed for CD8^+^ T cells (*p =* 0.031; Figure 2B data from seven patients available for analysis). Post-PIT CD8^+^ proliferation showed a greater reduction (mean pre-PIT to post-PIT CD8^+^ proliferation was reduced from 8.0% to 2.5% [pre-PIT range 1.8%–20.8%; post-PIT range 0.3%–4.7%]).

### Modulation of Cytokine Secretion following Peptide Immunotherapy

In order to characterise modulation of cytokine responses following PIT, culture supernatants were collected after 48 h. Cytokines were measured simultaneously by flow cytometry. The majority of patients displayed increased IL-10 secretion although this change did not achieve statistical significance. IL-5 secretion was significantly reduced post-PIT (*p =* 0.02; Table 1). IFN-γ secretion tended to be reduced following PIT, but heterogeneity was observed.

**Table 1:**
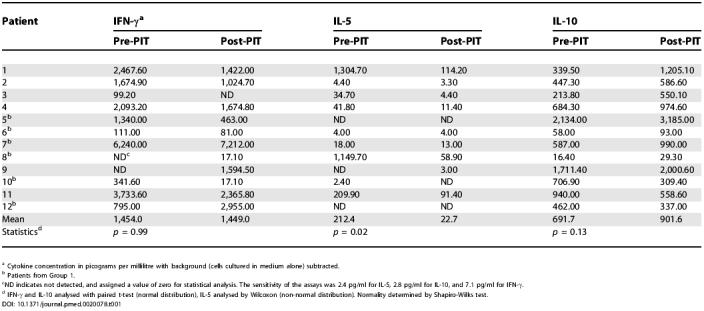
Modulation of Cytokine Secretion Profiles in Allergen-Stimulated PBMCs following PIT

^a^ Cytokine concentration in picograms per millilitre with background (cells cultured in medium alone) subtracted

^b^ Patients from Group 1

^c^ND indicates not detected, and assigned a value of zero for statistical analysis. The sensitivity of the assays was 2.4 pg/ml for IL-5, 2.8 pg/ml for IL-10, and 7.1 pg/ml for IFN-γ

^d^ IFN-γ and IL-10 analysed with paired t-test (normal distribution), IL-5 analysed by Wilcoxon (non-normal distribution). Normality determined by Shapiro-Wilks test

### PIT Leads to the Induction of a CD4^+^ T Cell Population with Suppressor Activity

**Figure 3:**
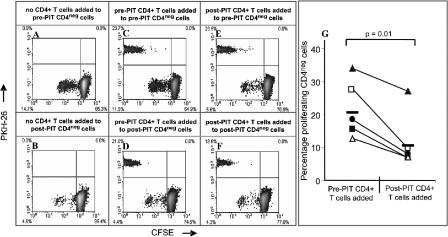
CD4^+^ Cells Isolated after PIT Suppress the Proliferative Response of Baseline CD4^neg^ Cells PBMCs taken before and after PIT were separated into CD4^+^ and CD4^neg^ populations by immunomagnetic separation. CD4^neg^ cells were labelled with CFSE and served as target cells. CD4^+^ cells were labelled with PKH-26 and were evaluated for suppressor/regulator function by co-culture with CD4^neg^ cells. (A) and (B) show antigen-stimulated proliferation of CD4^neg^ target cells before and after PIT. Proliferation of CD4^neg^ target cells was reduced after PIT (B). In (C) and (E), pre-PIT CD4^neg^ cells were employed as target cells. The addition of post-PIT (E), but not pre-PIT (C) CD4^+^ cells inhibited proliferation. In (D) and (F), post-PIT CD4^neg^ cells were employed as target cells. Addition of either pre-PIT (D) or post-PIT (F) CD4^+^ cells had no further effect on proliferation. Proliferation in the absence of a stimulus was less than 2% in all experiments. Representative data for one patient are shown. Data for an additional four patients were obtained using the same protocol. A data summary of percentage proliferation of pre-PIT CD4^neg^ PBMCs in the presence of pre-PIT or post-PIT CD4^+^ T cells for five patients from treatment Group 2 is shown in (G). The paired t-test was used for statistical analysis.

To identify populations of T cells with suppressive activity and to attempt to distinguish between active suppression and clonal deletion as potential mechanisms following PIT, pre- and post-PIT CD4^+^ T cells were isolated and their effect on proliferation of the CD4^neg^ fraction measured (Figure 3). Two distinct cell-cycle tracking dyes, CFSE and PKH-26, were employed to visually separate the target (CD4^neg^; CFSE) from the effector (CD4^+^; PKH-26) populations, by flow cytometry. PIT resulted in a 69% reduction (14.7% proliferation pre-PIT versus 4.6% proliferation post-PIT) in proliferation of the CD4^neg^ T cell population for the one representative patient shown in detail (Figure 3A and 3B). When pre- or post-PIT CD4^+^ T cells were added to CD4^neg^ PBMCs (at a ratio of 1:4), a marked reduction in proliferation of cat-allergen-specific CD4^neg^ pre-PIT T cells was observed when co-cultured with post-PIT (Figure 3E; 7.9% proliferation) but not with pre-PIT CD4^+^ T cells (Figure 3C; 17.7% proliferation), indicating that the post-PIT CD4^+^ T cells harboured a suppressor population. As post-PIT CD4^neg^ T cell proliferation was minimal, addition of post-PIT CD4^+^ T cells did not have a further suppressive effect on this cell population (Figure 3F). Additionally, removal of the CD4^+^ T cells from post-PIT PBMCs did not cause the depleted PBMC population to proliferate (Figure 3B), suggesting that antigen-specific cells in the post-treatment population had already been rendered anergic in vivo as a result of PIT, or possessed the ability to actively suppress responses themselves. Similar experiments were performed in a further four patients. A summary of the results for all five patients is shown in Figure 3G. Inhibition of proliferation by CD4^+^ post-PIT T cells ranged from 64.0% to 19.6%, with a mean of 47.5%.

### Phenotypic Characterisation of Candidate Regulatory T Cells Induced Post-PIT

**Figure 4:**
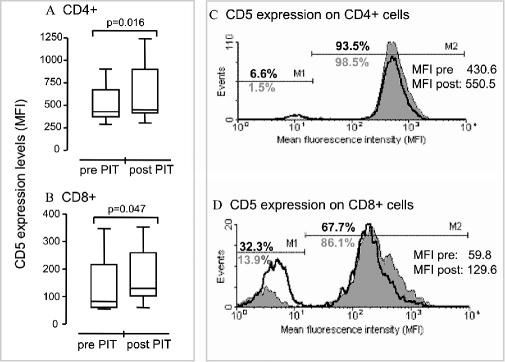
PIT Enhances CD5 Expression on Resting CD4^+^ and CD8^+^ PBMCs (A and B) Box-and-whiskers plots representing changes in MFI of CD5 expression levels on unstimulated CD4^+^ (A) and CD8^+^ (B) pre- and post-PIT PBMCs from seven patients in treatment Group 2. Isotype control MFI values have been subtracted. (C and D) CD5 levels on pre-PIT (heavy black line) and post-PIT (grey, filled) CD4^+^ and CD8^+^ PBMCs of one representative patient. M1 marks the CD5^low^ population, with a pre- to post-PIT decrease in CD5^low^CD4^+^ PBMCs from 6.6% (in black) to 1.5% (in grey), and a decrease in CD5^low^CD8^+^ PBMCs from 32.3% to 13.9%. M2 indicates the concomitant increases in CD5^+^CD4^+^ and CD5^+^CD8^+^ cells post-PIT. Changes in MFI values for the total pre- and post-PIT CD4^+^ or CD8^+^ populations of the single representative patient are shown in the upper right-hand corner of each histogram. The Wilcoxon signed rank test was used for statistical analysis.

T cell surface markers known to be associated with tolerance induction, such as CD25 and CD5, were compared on pre- and post-PIT resting PBMC in an attempt to provide further insight into the nature of the suppressor population. No significant variation was found in CD4^+^CD25^+^ cell numbers (mean pre-PIT to post-PIT proliferation 20.5%–17.9%; data not shown). However, when CD5 expression was determined on both CD4^+^ and CD8^+^ cells, a significant increase in MFI in both populations was observed (*p =* 0.016 and 0.047, respectively). Figure 4A and 4B show increases in CD5 expression on CD4^+^ and CD8^+^ cells (MFI of CD5 expression on CD4^+^ cells: pre-PIT mean, 465.5 [range, 290.3–908.5]; post-PIT mean, 559.7 [range, 302.5–1241.8]; range of post-PIT percentage change in MFI, 4%–37%; MFI of CD5 expression on CD8^+^ cells: pre-PIT mean, 110.9 [range, 55.3–345.8]; post-PIT mean, 149.2 [range, 60.6–352.2]; range of post-PIT percentage change in MFI, 7%–118%). Increased MFI resulted not only from a decrease in the numbers of CD5^low^ cells and an associated increase in CD5^+^ cells in both populations, but from an increase in CD5 expression on the CD5^+^ cells as well, as is shown in Figure 4C and 4D for one representative patient.

The effect of PIT on proliferation, cytokine secretion patterns, phenotype of T cell subsets, and suppressive capacity did not appear to be dependent on the total dose of peptide administered in the two treatment groups.

## Discussion

Following PIT, proliferation of CD4^+^, CD8^+^, and CD45RO^+^ memory T cells was reduced following culture with whole cat dander allergen extract. Non-specific T cell receptor (TCR) ligation with anti-CD3/CD28 was unaffected, implying that only cat-allergen-specific T cells had been targeted by PIT. The reduction in proliferation was primarily observed within the differentiated memory (CD45RO^+^) rather than the naïve T cell population, the latter displaying minimal cell division.

While the role of CD4^+^ T cells in the pathogenesis of allergic disease is well established, that of CD8^+^ T cells is less well defined. A number of reports suggest that CD8^+^ T cells may be activated in the asthma process. CD8^+^ cells from both bronchoalveolar lavage fluid and peripheral blood from atopic donors were found to produce IL-4 and IL-5 in lavage samples and bronchial biopsies [30,31]. Moreover, individuals with severe atopic disease have high frequencies of Dermatophagoides pteronyssinus 1–specific CD8^+^ T cells that secrete significantly more IL-4, IL-5, and IL-13 than non-atopic individuals [32]. Here we have shown that CD8^+^ T cells proliferate markedly to cat allergen in vitro even in the absence of CD4^+^ T cells. In the context of previous studies, it appears likely that these cells may contribute to disease pathogenesis. Thus, induction of non-responsiveness in CD8^+^ T cells should have a positive therapeutic outcome in allergic disease.

Cytokine profiles of cat-allergen-stimulated PBMCs were established following peptide therapy. Levels of IL-2 and IL-4 were generally below the limit of detection of the assays employed. PIT had no effect on secretion of tumour necrosis factor α (data not shown). Production of IL-5, a cytokine considered particularly relevant in asthma, was significantly reduced following PIT. In approximately half of the patients there were reductions in both Th1 and Th2 cytokines, as previously described [26]. We have observed similar results in an unpublished study of PIT for bee venom hypersensitivity. The majority of patients showed increased IL-10 production after PIT, in agreement with our earlier observations. However, in the present study this did not achieve statistical significance, in contrast to a previous report. Enhanced production of IL-10 has been associated with protection from allergic symptoms in both naturally exposed individuals such as beekeepers and in individuals receiving bee venom immunotherapy [33]. In contrast, IL-10 production in relation to cat allergen exposure and protection is less well established. A protective effect of high-dose natural exposure to cat allergens (resulting in a “modified Th2 response”) has been reported [34,35]. Woodfolk and colleagues demonstrated elevated IL-10 production in individuals displaying a “modified Th2” profile in which cat-allergen-specific IgG4 appeared to protect from disease [36]. In their study, particular regions of the Fel d 1 molecule (carboxy terminus of chain 2) appeared to be associated with presentation by HLA-DRB1*0701 and were associated with preferential IL-10 induction. For technical reasons, peptides from this region were not included in the preparation used in the present study. Inclusion of such peptides in future studies may enhance vaccine efficacy.

In the present study, cytokine production was evaluated in peripheral blood cells. Cytokine production at local tissues targeted by allergens may provide a more accurate picture of the effects of immunotherapy with peptides or native allergens/allergen extracts. For example, in a related study a significant increase in the number of cutaneous CD4^+^IFNγ ^+^ cells (*p =* 0.03), but not in CD4^+^IL-10^+^ cells, was observed in allergen-challenged skin biopsies [27]. Similarly, a significant increase in the number of IFN-γ mRNA(+) cells (*p* = 0.03) was found in nasal biopsies of patients enrolled in a whole-grass-pollen immunotherapy trial, in the absence of significant modifications in IFN-γ secretion by corresponding in vitro stimulated PBMCs [37]. Thus, in vivo localization of cells by allergen challenge may reveal patterns of immunomodulation that differ from changes in the blood of the same individual. For this reason, caution should be exercised when interpreting alterations in cytokine profiles in different tissues following immunotherapy.

We addressed the possibility that changes in T cell proliferation and cytokine secretion may be related to the induction of a population of T_reg_ or suppressor T cells, similar to that observed following peptide intervention in murine models [38]. PBMCs were separated into CD4^+^ and CD4^neg^ populations. CD4^+^ cells isolated from post-PIT blood were able to actively suppress the proliferation of pre-treatment CD4^neg^ cells. The selection of CD4^neg^ cells, rather than CD4^+^ cells, as targets was due to limitations in the number of cells available. Nevertheless, the results obtained indicate the induction of regulatory and suppressor CD4^+^ T cells following PIT. Interestingly, removal of CD4^+^ cells from the post-PIT PBMC population did not lead to a reversal of the allergen-specific hyporesponsiveness in the pre-PIT CD4^neg^ population. This observation suggests that in addition to active suppression by CD4^+^ cells, enduring effects of therapy can also be detected. Explanations for such observations may include the following: (i) clonal deletion of some antigen-specific CD4^neg^ cells, (ii) the induction of anergy in these cells during the treatment phase, or (iii) the presence of a CD4^neg^ suppressor population. In support of the last possibility, regulatory CD8^+^ T cells have recently been described [39]. Studies identifying allergen-specific CD4 and CD8 T cells will be required to address such issues. In future studies it will be of interest to identify the subpopulation or subpopulations of CD4 and CD8 cells responsible for the suppressive effect, by removing candidate T cells from the pre- and post-treatment CD4^+^ T cell populations prior to co-culture.

No increase in numbers of CD4^+^CD25^+^ cells was observed in PBMCs following PIT, in contrast to studies of whole allergen immunotherapy [40,41]. In fact, numbers of CD25^bright^ cells significantly decreased following peptide therapy (data not shown). Furthermore, CD4^+^CD25^+^ T cells obtained before and after treatment in a related PIT study did not differ in their ability to suppress allergen-specific effector T cell proliferation and IL-13 production, arguing against a major role for this type of regulatory cell in peptide therapy [42]. We speculate that PIT results in T cell activation in the absence of inflammatory signals, possibly via presentation by immature APCs, or even by neighbouring T cells. Well-characterised in vitro human models have demonstrated that it is indeed possible to induce T cell anergy following incubation with cognate peptide in the absence of professional APCs [43,44]. Recently, Apostolou and von Boehmer reported induction of antigen-specific hyporesponsiveness, mediated by regulatory cells, following continuous, low-dose peptide administration in mice [21], an observation that supports our current and previous clinical findings. Additionally, Prakken and colleagues have demonstrated induction of IL-10-secreting T_reg_ following oral peptide therapy in patients with rheumatoid arthritis. These cells may similarly represent an induced population of adaptive T_reg_ [45].

While CD25 expression is considered to be a marker of a functionally distinct population of T_reg_ (provided the cells have not been recently activated), CD5 expression levels on T cells may be an indicator of a regulatory function [18]. CD5 has been shown to be a negative regulator of TCR signalling, influencing the fate of developing thymocytes [17]. In the periphery, CD5^neg^ T cells show enhanced proliferation to TCR triggering [46]. Conversely, increased membrane levels of CD5 correlate with a lowering of the T cell response to antigen by targeting downstream signalling events [47].

In the current study, CD5 levels were significantly elevated on directly ex vivo, unstimulated CD4^+^ and CD8^+^ T cells, following peptide therapy. The increases were slight, which likely relates to the low precursor frequency of the cells targeted. Interestingly, the increases observed on CD8^+^ T cells were partly due to a reduction in the size of the CD8^+^CD5^neg^ T cell population. A distinct CD8^+^CD5^neg^ T cell population that accounts for 3%–10% of the total CD8^+^ T cell population in healthy donors has previously been described [48] and appears to be the main producer of lymphotactin (XCL-1) [49]. As the average size of CD8^+^CD5^neg^ T cell populations in the allergic asthmatic patients in our study is substantially larger (23.2% of the total CD8^+^ T cells), it is tempting to speculate that this is further evidence for a dysregulated immune response associated with allergic disease. This observation is in agreement with data from lymphopenic mice that developed wasting disease with accelerated kinetics following adoptive transfer of T cells expressing low levels of CD5, whilst CD5^hi^ cells were protective [18]. Consistent with these findings, surface levels of CD5 on human T cells also appear to correlate with immune function, as the CD5^neg^ population was increased in bone marrow transplant recipients as well as in patients with advanced AIDS [50,51]. However, as relatively little is known about the role of CD5 in human T cell tolerance, further investigations are required to establish the relevance of our finding in allergic disease. Isolating Fel d 1–specific T cells should yield valuable information on the functional relevance of increased CD5 expression on allergen-specific cells.

To our knowledge, this is the first demonstration that PIT induces a CD4^+^ T cell population that actively suppresses antigen-induced proliferation of effector T cells. The use of dual labelling with distinctly coloured dyes allowed evaluation of the effect of the CD4^+^ T cell subset on the proliferation of CD4^neg^ (including CD8^+^ cells, natural killer cells, B cells, monocytes, and basophils) using flow cytometry. While dual labelling has been widely used to track cell migration in animal models [52], its application in in vitro human T cell proliferation experiments has, to our knowledge, not previously been reported. Single-colour labelling of distinct human PBMC populations has been used to characterise, isolate, and clone peanut-allergen-specific T cells [53] and to determine precursor frequencies of recall-antigen-specific T cells [54]. Measurement of proliferation by means of CFSE has the additional advantages of requiring relatively low numbers of cells and allowing additional phenotypic (cell surface markers) or functional parameters (intracellular cytokine secretion) to be studied in parallel, in distinct subpopulations [54].

In conclusion, our data indicate that low-dose PIT targets both CD4^+^ and CD8^+^ memory T cells and induces a population of active suppressor/regulatory T cells within the CD4^+^ compartment. Suppressor activity may also reside within the CD4^neg^ compartment. Peptide therapy resulted in a heterogeneous modulation of allergen-specific PBMC cytokine responses in vitro, generally characterised by IL-10 induction and IL-5 suppression. Finally, modest but consistent increases were observed in surface CD5 expression on both CD4^+^ and CD8^+^ T cells, an observation that may be linked to the induction of antigen-specific hyporesponsiveness. The ability to modulate antigen-specific T cell function in vivo has important implications for the treatment and prevention of allergic, autoimmune, and allograft-related diseases.

## Supporting Information

**Accession Numbers**

The SwissProt (http://www.ebi.ac.uk/swissprot/) accession numbers for the gene products discussed in this paper are CD5 (P06127), DEC-205 (Q60767), Fel d 1 chain 1 (P30438), Fel d 1 chain 2 (P30440), and lymphotactin (P47992).

**Patient Summary**

**Background**

Increasing numbers of people are developing allergies to pets and becoming asthmatic. It is not clear what combination of events triggers allergy—for example, whether keeping pets as a child is protective—nor what can be done to treat the allergy once it develops.

**What Did the Authors Do?**

They looked at a small group of people who were allergic to cats and asthmatic. They measured the levels of different kinds of T cells in their blood—cells that are associated with the allergy. They then treated the people with small proteins that are very similar to the triggers for the allergy and looked to see how the levels of various T cells changed. They found that the protein treatment triggered a particular type of cell, which seemed able to repress the reactive cells that had triggered the immune reaction previously.

**What Do These Results Mean for Patients?**

There are many things that interact to produce allergy, and this study does not help in understanding exactly how this happens. It does suggest a way that treatment with specific small proteins might work in reducing the allergy; however, the results will need to be confirmed in much larger studies.

**Where Can I Get More Information?**

Both the American Academy of Allergy Asthma and Immunology, and Asthma UK have large sections of patient information: http://www.aaaai.org/patients.stm; http://www.asthma.org.uk/

## Abbreviations

APC: antigen-presenting cell
CFSE: carboxyfluorescein diacetate succinimidyl ester
DC: dendritic cell
Fel d 1: Felis domesticus allergen 1
IFN: interferon
IL: interleukin
MFI: mean fluorescence intensity
PBMC: peripheral blood mononuclear cell
PIT: peptide immunotherapy
TCR: T cell receptor
Th: T helper
T_reg_: regulatory T cell(s)
